# Quantifying the effects of achilles tendon lengthening surgery: An intraoperative approach

**DOI:** 10.3389/fphys.2023.1143292

**Published:** 2023-03-06

**Authors:** Elena Brendecke, Stefanos Tsitlakidis, Marco Götze, Sébastien Hagmann, Filiz Ates

**Affiliations:** ^1^ Clinic of Orthopedics and Trauma Surgery, Heidelberg University Hospital, Heidelberg, Germany; ^2^ Institute of Structural Mechanics and Dynamics in Aerospace Engineering, University of Stuttgart, Stuttgart, Germany

**Keywords:** triceps surae, idiopathic foot deformity, *in vivo* muscle mechanics, muscle lengthening surgery, cerebral palsy

## Abstract

Achilles tendon lengthening (ATL) is frequently used in the treatment of foot deformities. However, there is currently no objective method to determine the optimal muscle length during surgery. We developed an intraoperative approach to evaluate the passive and active forces of the triceps surae muscle group before and after ATL and aimed to test the following hypotheses: 1) the ankle passive range of motion (ROM) increases, 2) passive muscle forces decrease post-ATL, and 3) forces measured from patients with non-neurological and neurological conditions demonstrate different characteristics. Passive forces at various ankle joint positions were measured in ten patients (11.3 ± 3.0 years old) pre- and post-ATL using a force transducer attached to the Achilles tendon. In six patients, active isometric forces were measured by stimulating the triceps surae supramaximally. Passive forces decreased by 94.3% (*p* < 0.0001), and ROM increased by 89.4% (*p* < 0.0001) post-ATL. The pre-ATL passive forces were 70.8% ± 15.1% lower in patients with idiopathic foot deformities than in patients with neurological conditions (*p* < 0.001). The peak active force of 209.8 ± 114.3 N was achieved at an ankle angle of 38.3° ± 16.0°, where the passive force was 6.3 ± 6.7 N. The inter-individual variability was substantial in both groups. In conclusion, the hypotheses posed were supported. The present findings suggest that muscle passive and active force production as well as the inter-individual variability should be considered when planning further treatment.

## Introduction

Skeletal muscles need continuous stretching impulses to grow and develop properly (Huijing and Jaspers, 2005). If this growth stimulus is disrupted by involuntary and prolonged muscle contractions, permanent muscle shortening can occur, resulting in bone and joint deformities. One cause of contraction formation is spasticity, often observed in patients with different underlying neurological conditions such as cerebral palsy (CP). These velocity-dependent repetitive muscle contractions can lead to limitations in force exertion and joint range of motion (ROM) (Farmer and James, 2001; Wren et al., 2005). In the lower limb, triceps surae muscle spasticity resulting in foot deformities such as pes equinus or pes equinovarus are common manifestations (Horsch et al., 2019; Bloom and Sabharwal, 2022) with possible deterioration of the patients’ ability to stand or walk independently (Prabhu et al., 2013; Attias et al., 2016). Muscle shortening and associated joint movement problems are not limited to patients with spastic CP and neurological conditions. Foot deformities due to permanent contractures can occur without neurological origin (Engström and Tedroff, 2018). Pes equinus appears in toe-walking children and the majority of patients with congenital talipes equinovarus (CTEV) are considered idiopathic (Ruzbarsky et al., 2016). Both idiopathic and neurological forms may require surgical intervention but the approach and clinical outcome differ (Gurnett et al., 2008; Brierty et al., 2021). The exact definition of a functionally relevant and impairing equinus foot is still controversial (Horsch et al., 2019; Horsch et al., 2022).

Achilles tendon lengthening (ATL) is a frequently used component in the treatment of pes equinus and recurrent CTEV (Yngve and Chambers, 1996; Rutz et al., 2020). This surgical procedure aims to increase the ROM and improve the patient’s ability to stand and walk by enabling the patient to roll over their feet from heel to toe (Sutherland and Cooper, 1978; Dietz et al., 2006). There are several methods to lengthen the Achilles tendon and the choice depends on the amount of lengthening required, clinical examination, and underlying disease (Dietz et al., 2006; Rutz et al., 2020).

Currently, there is no objective method to quantify the amount of tendon lengthening required to reduce tension without critical muscle weakening. Instead, surgeons rely on their haptic impressions and intuition to determine “the dose of lengthening”. Thus, the risk of over- or under-correction is not negligible (Borton et al., 2001; Dietz et al., 2006; Firth et al., 2013). A few preoperative methods exist to quantify the needed amount of lengthening (Pilloni et al., 2019; Ozyalvac et al., 2020). Yet, these indirect methods are limited in terms of estimating the outcome. Therefore, there is a need for a direct approach that would give surgeons immediate objective feedback during surgery. In this study, we aimed to investigate the use of an intraoperative approach (e.g., Ateş et al., 2013; Ates et al., 2014, 2016; Yucesoy et al., 2017; Ates et al., 2018; Kaya et al., 2018) to measure the passive and active isometric forces of the triceps surae muscle group in relation to different ankle joint positions as well as quantify the effects of Achilles tendon lengthening (ATL) surgery. We hypothesized that i) the maximum dorsiflexion (DF) angle and passive ankle ROM increase, ii) passive muscle forces decrease significantly post-ATL, and iii) muscle forces measured from patients with the neurological disease are different from patients with idiopathic foot deformities.

## Materials and methods

### Patients

Ten patients (11.3 ± 3.0 years old, average body weight = 51.2 ± 22.2 kg at the time of surgery) who underwent surgery for their foot deformity after indication in our hospital’s pediatric orthopedic outpatient clinic were included. Five of the patients had an idiopathic foot deformity without a diagnosed neurological disease (here referred to as “non-neurologic”), five had a diagnosed underlying disease (four cerebral palsy, one HMSN Type IV = hereditary motor and sensory neuropathy, type IV = Refsum disease). Before the experiments, the patients and/or their parents or legal guardians provided written informed consent. The anthropometric data were collected (Table 1).

**TABLE 1:** Patient characteristics.

#	Diagnosis	Age (y/m)	Sex	Foot deformity	Leg length (cm)	Lower leg length (cm)	Foot length (cm)
**1**	Cerebral palsy	12/0	F	Pes equinus	88	40	24
**2**	HMSN	13/8	M	Pes equinus	86	36	19.5
**3**	Cerebral palsy	12/4	F	CTEV	81	37	19.5
**4**	Cerebral palsy	10/4	M	Pes equinus	55	24	17
**5**	Cerebral palsy	14/5	M	Pes equinus	76	32	27
**6**	Non-neurologic	10/4	F	Pes equinus	86	38	22
**7**	Non-neurologic	6/9	F	CTEV	58	24	17
**8**	Non-neurologic	6/3	M	CTEV	60	29	14
**9**	Non-neurologic	16/10	M	Pes equinus	98.5	45	26
**10**	Non-neurologic	14/0	M	Pes equinus	88.5	40	22

y = years, m = months, HMSN = hereditary motor and sensory neuropathy, CTEV = congenital talipes equinovarus.

### Intraoperative procedures

**FIGURE 1:**
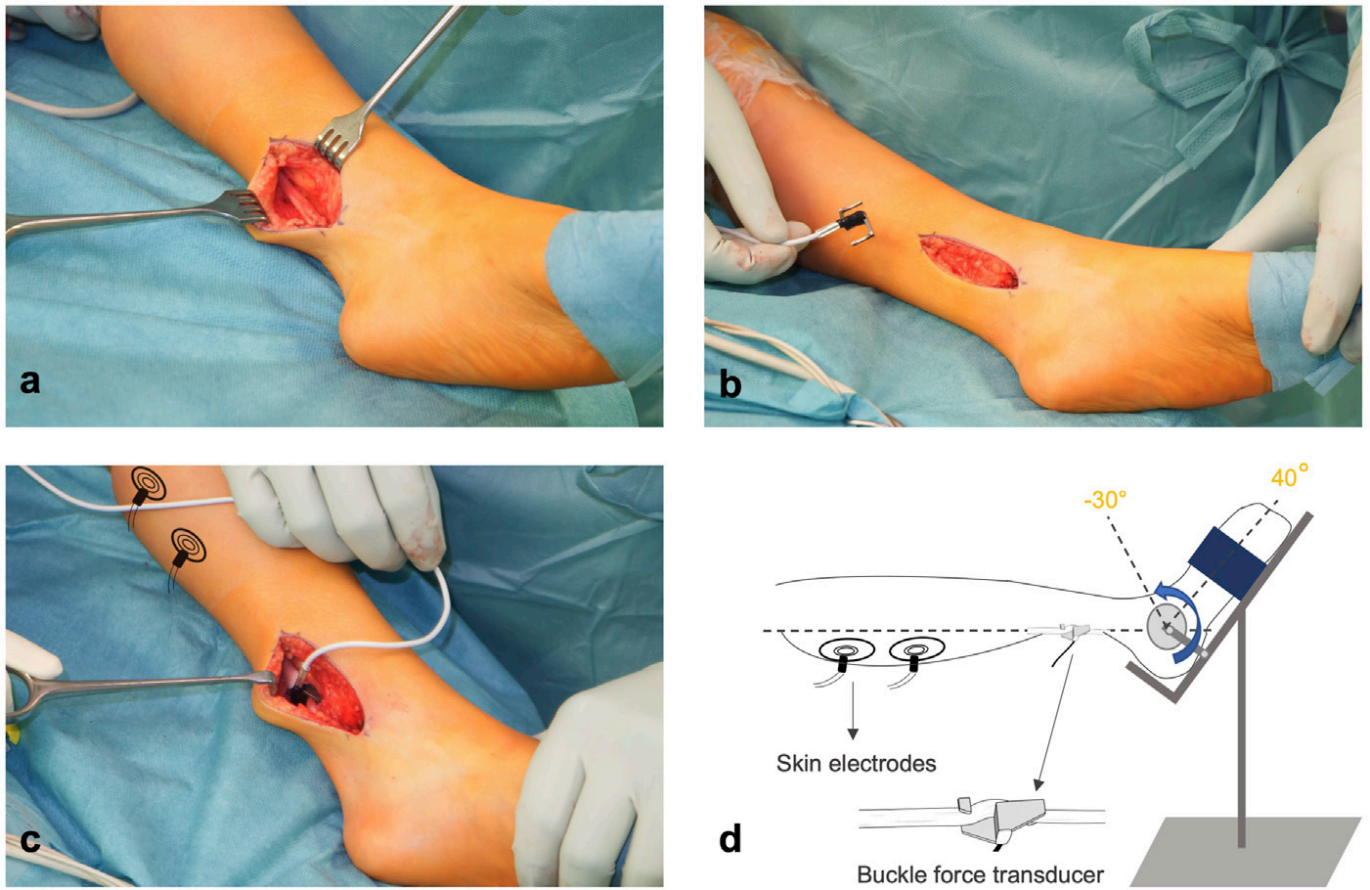
Intraoperative experimental setup. **(A)** Achilles Tendon is exposed under general anesthesia and **(B,C)** the s-shaped buckle force transducer is attached. **(D)** Two electrodes are placed on the skin over the triceps surae muscle for active force measurement. The foot is mounted on an apparatus with a heavy ground plate and an ankle angle adjuster. The foot is fixed in the apparatus to collect isometric force data.

The experimental procedures were approved by the local ethics committee. All procedures were performed in agreement with the guidelines of the Helsinki declaration. The patients received an Achilles tendon lengthening (ATL) performed as a Z-plasty (Yngve and Chambers, 1996) under general anesthesia. The Achilles tendon was made visible with a 6–7 cm posteromedial incision. The tendon was exposed from the calcaneus (insertion) to the musculotendinous junction. Before any other surgical intervention was performed, a sterilized s-shaped buckle force transducer (BFT) (dimensions: width = 12 mm, length = 20 mm, and height = 9 mm; maximal force range = 500 N; for test range 0–200 N: accuracy <3% (<0.2% below 100 N); resolution = 0.6 N and high linearity (*R* ^2^ = 0.99, peak non-linearity = 1.3%)) was mounted onto the tendon and secured (Figure 1). Prior to use, BFTs were calibrated using bovine tendon strips and sterilized. The BFT was then connected to an amplifier (NI 9237, National Instruments, USA) and a data acquisition system (NI cDAQ-9174, National Instruments, USA).

For active muscle force measurements, two sterilized skin electrodes (standard ECG electrodes) were placed on the gastrocnemii muscle bellies and connected to a constant current high voltage source (cccVBioS, TEKNOFIL, Istanbul, Turkey). The patient’s foot was mounted on the device (Figure 1D) designed to fix the foot at the adjusted ankle positions. This device included a heavy ground plate to prevent movement during active force measurements. An attached goniometer allowed a precise angle adjustment. The center of foot rotation and the apparatus were carefully aligned.

### Collection of passive and active forces

*Preconditioning:* The triceps surae muscle group was preconditioned by activating with a supramaximal transcutaneous electrical stimulation (with a bipolar rectangular signal, 200 mA, 50 Hz) at the longest and the shortest possible muscle lengths -corresponding to the patient’s maximal plantarflexion (PF) and dorsiflexion (DF) respectively-repeatedly until the measured active forces did not change at the measured position.

**FIGURE 2:**
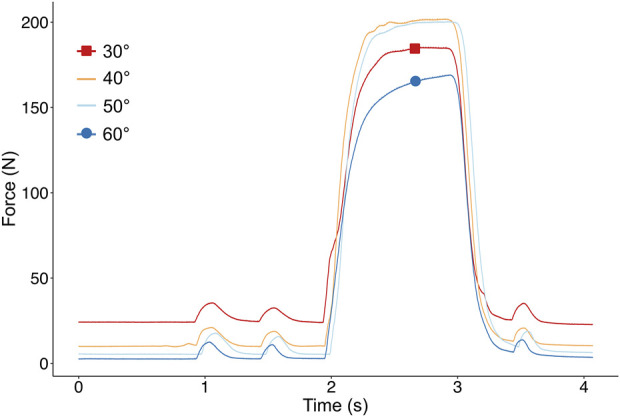
Example of superimposed force-time traces collected from the Achilles Tendon of one patient at different ankle angles. After two pulsatile stimulations, the muscle is stimulated supramaximally to reach a tetanic plateau and the maximal muscle force.

*Pre-ATL:* Triceps surae was activated with two short pulses followed by a pulse train for 1,000 ms to induce tetanic contraction and a subsequent twitch. BFT recorded passive and active isometric forces from the Achilles tendon at fixed positions of ankle angle from the patient’s maximal PF to DF in steps of 10°. Figure 2 shows the superimposed examples of force-time traces collected from one patient’s triceps surae muscle group at different ankle angles. After each contraction, the muscle was given a 2-min break to recover.

After pre-ATL measurements were completed, the BFT was removed and the surgeon performed the Z-plasty: The tendon was divided by a vertical incision using a scalpel. After reaching the desired length, the scalpel was turned by 90° to cut through half of the tendon. The remaining half was cut on the opposite end of the vertical section to create a Z-shaped incision. After applying the correction, the two free ends of the tendon were then sutured with a modified Bunnell technique (Bunnell, 1922) using an absorbable, braided suture (Ethicon, Vicryl 1, Polyglactin 910, Johnson and Johnson, New Brunswick, New Jersey, USA) while the patient’s foot was held in a plantigrade position (0°).

*Post-ATL:* After completing the Z-plasty, the BFT was mounted onto the tendon again. The passive forces in this new condition were measured throughout the full ROM. The still fragile sutures made post-ATL active measurements impossible.

### Data processing and statistics

Passive force (F_p_) was averaged over a period of 1,000 ms and fitted in relation to the ankle angle (AA) with an exponential function using a least squares criterion:

$$F_{P}=e^{a_{0}+a_{1}x}+a_{2}$$

a_0_, a_1_, and a_2_ are the coefficients determined during the fitting process. A linear function was used if the exponential function did not describe the collected passive force-ankle angle data.

Active force (F_A_) -calculated by subtracting passive muscle force (F_P_) from total muscle force-was averaged over a 400 ms period within the tetanic plateau and fitted with a polynomial function using a least squares criterion:

$$F_{A}=a_{0}+a_{1}AA+a_{2}{AA}^{2}+\ldots +a_{n}{AA}^{n}$$

a_0_, a_1_, a_2_, and a_n_ are the coefficients determined in the fitting process, AA = ankle angle. The degree of the polynomial function was chosen based on the best-fit criteria so that the fitted function adequately described the particular set of force-angle data. The fitted force data were used to determine the mean passive and total muscle forces for each ankle angle as well as the peak muscle force and its corresponding angle.

To evaluate force production capacity independent from muscle length, passive and active forces were normalized to the lower leg length of individual patients.

Student’s t-test was used to compare the maximum DF and ROM before and after the ATL. Two-way ANOVA for repeated measures (factors: pre-vs post-ATL; ankle angle) was applied to compare 1) pre- and post-ATL passive forces and 2) the passive muscle forces between neurological and non-neurological patients. Post-hoc tests for pairwise comparisons were applied to locate the differences. Differences were considered significant at *p* < 0.05.

## Results

**FIGURE 3:**
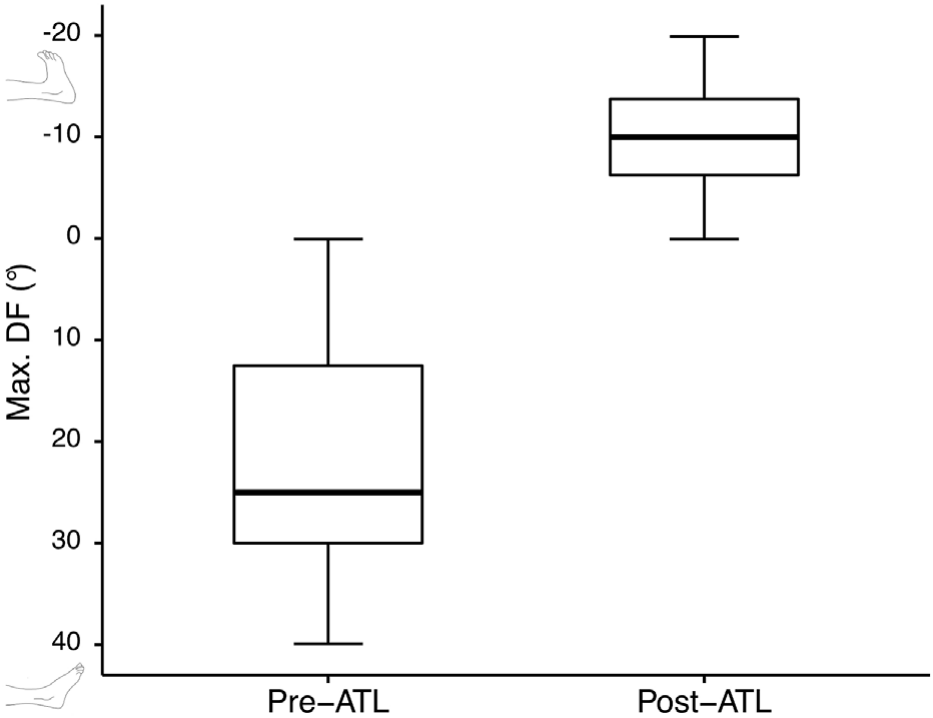
The maximal dorsiflexion pre- and post-ATL, measured passively under general anesthesia.

The patients’ ROM pre- and post-ATL are summarized in Table 2. The post-ATL ROM (62.5° ± 9.6°) was 89.4% higher than the pre-ATL ROM (33.0° ± 9.0°) (*p* < 0.0001). The increase in ROM was due to the change in maximum DF, as maximum PF remained unaffected. The mean maximum DF increased by 27.1% from 19° ± 13.0° pre-ATL to −10.5° ± 5.2° post-ATL (*p* < 0.0001) (Figure 3).

**TABLE 2:** Ankle passive range of motion measured pre- and post-ATL.

#	Max. PF (°)	Max. DF pre (°)	Max. DF post (°)	ROM pre (°)	ROM post (°)
**1**	50	0	−10	50	60
**2**	60	40	−10	20	70
**3**	60	30	−20	30	80
**4**	50	10	−15	40	65
**5**	50	30	0	20	50
**6**	60	30	−10	30	70
**7**	40	0	−10	40	50
**8**	50	20	−5	30	55
**9**	40	10	−15	30	55
**10**	60	20	−10	40	70

Max. = maximal, PF = plantarflexion, DF = dorsiflexion, ROM = range of motion, Pre = before ATL, Post = after ATL, Positive and negative ankle angle values refer to PF and DF positions, respectively.

**FIGURE 4:**
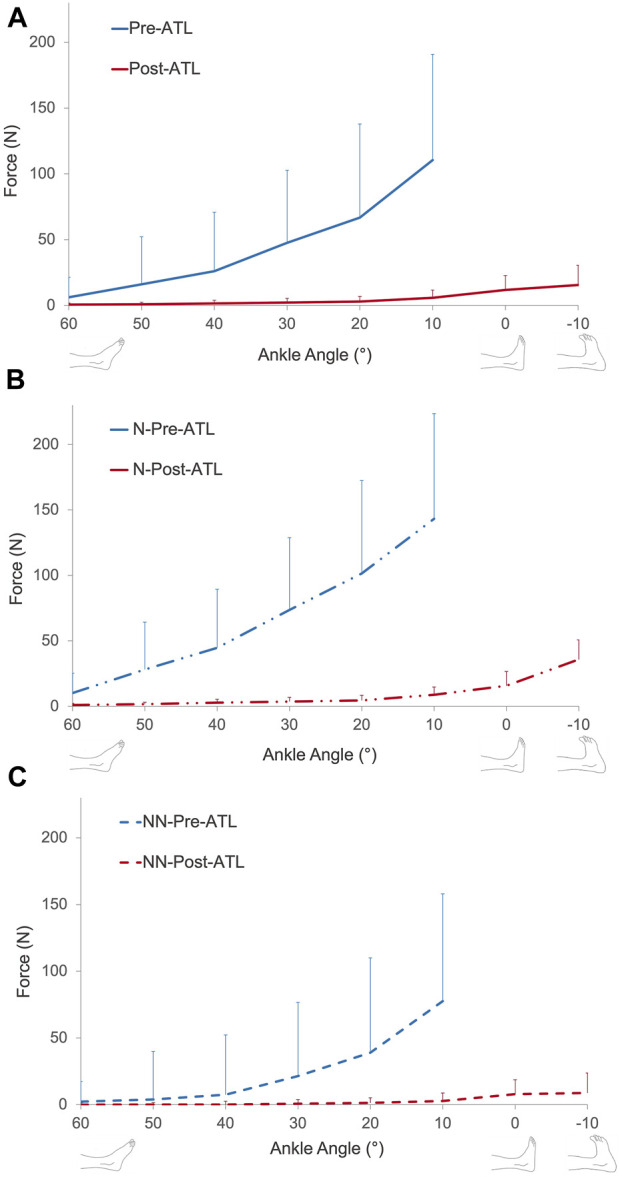
**(A)** Muscle passive force-ankle angle characteristics of all patients pre- and post-ATL. Muscle passive force-ankle angle characteristics of patients with **(B)** and without **(C)** neurological conditions pre- and post-ATL. N = neurologic patient, NN = non-neurological condition.

Passive forces showed an exponential increase with increasing muscle length. Pre- and post-ATL passive forces were averaged for all ten patients (Figure 4A). ANOVA showed significant effects for both factors (pre- and post-ATL forces (*p* < 0.0001) and ankle angle (*p* < 0.001)) with no significant interactions. Post-ATL passive force drop ranged from 90.4% to 95.6% at the measured joint positions; on average 94.3% ± 2.0%, indicating an absolute force reduction of 42.5 N (95% conf. int. = −56.2 to −28.7 N). For normalized passive forces, ANOVA showed significant effects of both factors (pre- and post-ATL forces (*p* < 0.0001) and ankle angle (*p* < 0.001)) with no significant interactions.

**FIGURE 5:**
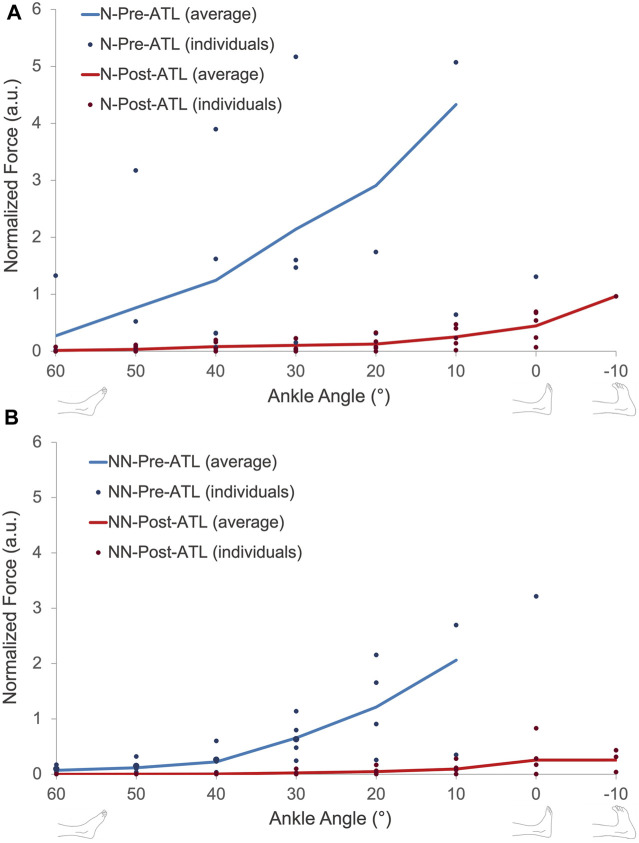
Normalized passive force-ankle angle characteristics of patients with **(A)** and without **(B)** neurological conditions pre- and post-ATL. N = neurologic patient, NN = non-neurological condition.

When patients were classified according to their neurological condition (Figures 4B, C), the pre-ATL passive forces were 45.8%–86.1% (at different joint positions) lower for patients with a non-neurological condition compared to the patients with neurological disease (*p* = 0.0016). The mean difference of −70.8% ± 15.1% corresponded to an absolute value of −43.0 N (95% conf. int. = −17.2 N to −68.0 N). The difference between the two groups (−77.2% ± 18.5%) remained after the ATL (*p* < 0.01). However, the absolute difference (−3.5 N, 95% conf. int. −1.1 N to −5.9 N) was less pronounced. Normalization did not cause a major change in comparison: The normalized passive forces pre-ATL were 52.3%–84.4% (at different joint positions) lower for patients with a non-neurological condition compared to the patients with neurological disease (Figure 5, *p* = 0.0013). Post-ATL, the difference in normalized forces remained (−82.5% ± 17.1%) between the two groups (*p* < 0.01).

**FIGURE 6:**
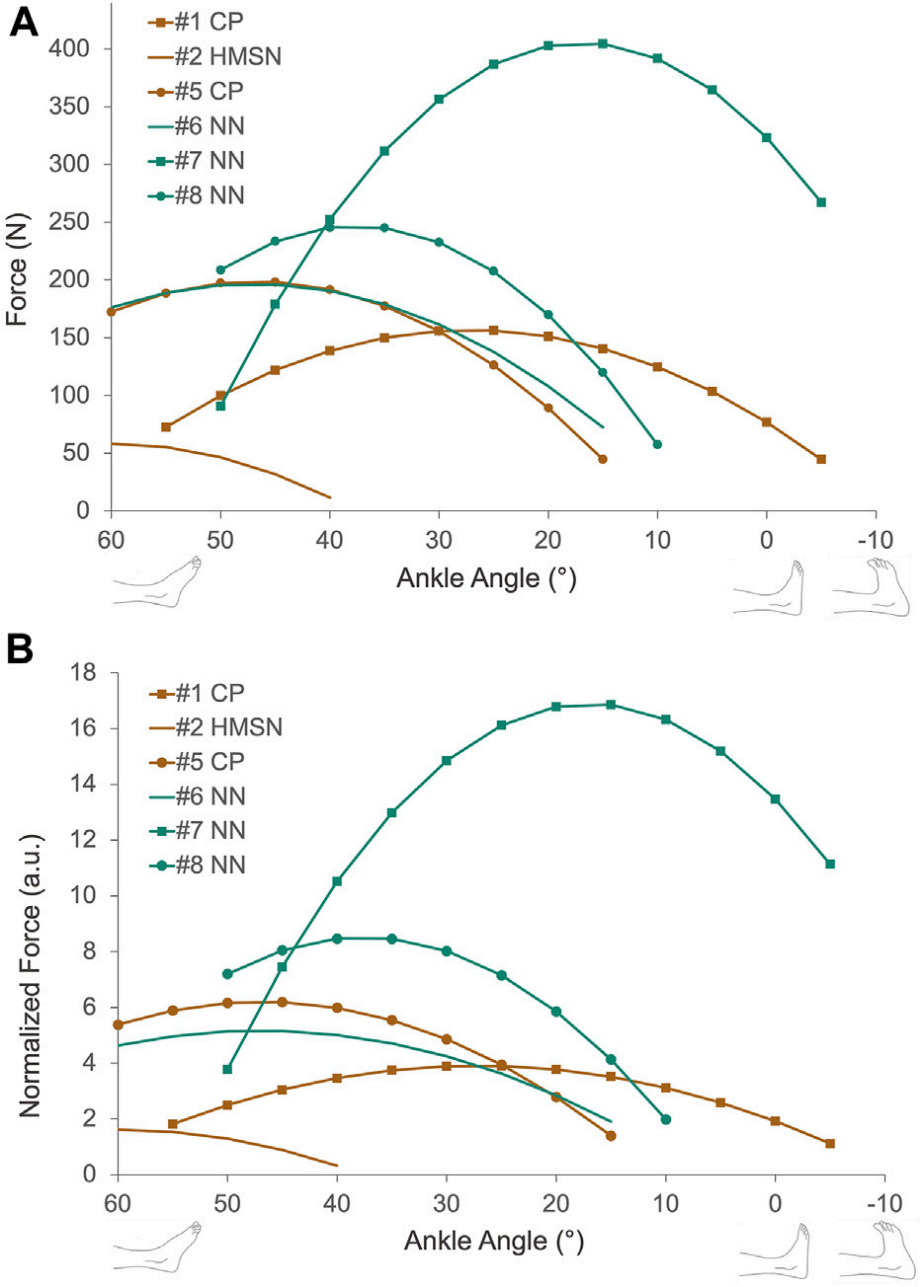
**(A)** Muscle active force-ankle-angle characteristics collected from six patients pre-ATL. Active force is calculated by subtracting passive force from the total measured force. **(B)** Normalized active force-ankle-angle characteristics pre-ATL. Active forces were normalized to the patient’s lower leg length. CP = Cerebral palsy, HMSN = hereditary motor and sensory neuropathy, NN = non-neurological condition.

We were able to measure the active force production of the triceps surae muscle group for six of the patients (Figure 6A). The peak value of active force production was 209.8 ± 114.3 N, ranges between 58.3 N and 404.5 N. Maximal active force production was achieved at 25°, 60°, 45°, 15°, 40°, and 45° PF angles (38.3° ± 16.0°) and the corresponding passive forces were 7.2 N, 0.7 N, 5.6 N, 19.0 N, 0.8 N, and 4 N (6.3 ± 6.7 N). Normalization of active forces pre-ATL did not cause any major change in the force production characteristics of patients tested (Figure 6B).

## Discussion

The main objective of the ATL procedure is to increase the ankle ROM and the length range of muscle force exertion. The surgery aims to attain at least a plantigrade foot position (0°) to improve the function (Dietz et al., 2006). Our present results show that these were achieved for all patients tested. Muscle-lengthening interventions were also expected to reduce passive and active muscle forces (Ates et al., 2013). Consistent with the expectation in passive state, we found a significant decrease in passive muscle forces. Therefore, the hypotheses posed were supported. The observed differences between the muscles of patients with neurological and non-neurological conditions and the possible alterations in active force production are discussed in the following sections.

### Passive muscle forces

Pre- and post-ATL passive forces showed a linear to exponential increase from DF to PF joint positions, corresponding to the increasing lengths of the triceps surae muscle group. The passive mechanical characteristics are consistent with previous findings in animal (Gordon et al., 1966; Ter Keurs et al., 1978) and human muscles (Rassier et al., 1999; Kreulen and Smeulders, 2008; de Bruin et al., 2014). After the ATL, the targeted reduction in passive muscle forces was achieved in both groups and this facilitated medium passive tension to shift approximately 30° toward the DF position.

Importantly, even though the present findings were statistically meaningful, the observed inter-subject variability was remarkable pre-ATL. Previous studies investigating exclusively patients with CP have also reported great variability (Smeulders et al., 2004; Ates et al., 2014; Yucesoy et al., 2017). The severity of the disease, differences in treatment approaches, and difficulties in the fine description of the severity may be some of the reasons. Differences in etiologies, particularly in patients with non-neurological conditions, may also explain the variability in part: Idiopathic foot deformities were classified as “non-neurological”, but the exact pathophysiology of the contractures remains unknown and may vary (Gurnett et al., 2008). In patients with CP, muscular changes such as a decrease in muscle volume and length (Barber et al., 2012), and an increase in connective tissue (Booth et al., 2001; de Bruin et al., 2014) have been reported as a result of disruption in the neural signal and abnormality in motor control (Ates et al., 2020). We found that inter-individual variance persisted even after normalization of the forces to the muscle length (Figure 5). Presently, there was no muscle volume (or thickness and physiological cross-sectional area) information collected for the patients recruited, so it is not possible to entirely rule out the effect of muscle volume in variance between individuals. However, muscle volume is expected to determine the force amplitude since it is associated with the amount of active force production but not necessarily the ankle angle range where forces are generated. Thus, the normalization of muscle volume may not cause dramatic shape changes in the mechanical characteristics which highlight possible differential changes in the intrinsic muscle properties rather than muscle size as adaptation factors of muscle passive force-ankle angle characteristics. Sarcomeric protein titin has a key role in myofilament integrity (Swist et al., 2020) and passive resistance where its two regions, corresponding to the I-band of a sarcomere, Ig domains, and PEVK were reported to show different mechanical properties; of the former being stiffer in moderate sarcomere stretches and latter being stiffer at higher stretches (Linke and Granzier, 1998). The amount and material properties of sarcomeric titin may alter in various conditions (e.g., van der Pijl et al., 2020). An earlier study reported that titin mass measured for gastrocnemius and soleus muscles was greater for the patients with CP compared to the typically developing children. (Mathewson et al., 2014). However, the authors could not find a correlation between titin size and muscle fiber passive stiffness. Importantly, this previous study also emphasized the heterogeneity of the data collected from the patients with CP being much higher. Together with these, our findings suggest that the mechanical adaptation of myofibers is not well-understood and needs more detailed investigations. It should also be noted that although titin is not a contractile element, it might as well affect the active muscle characteristics since titin is known to signal the active force production hence, regulating the muscle force exertion depending on sarcomere length (Linke et al., 2002; Rode et al., 2009; Herzog et al., 2012). Yet, the need for relating structural changes of sarcomeric proteins and intramuscular connective tissues with the force production and the resultant mechanical effects of tendon surgery is valid. There is also a data scarcity for patients with idiopathic foot deformities. The factors other than neurological conditions in the development of muscle shortening (Kruse et al., 2021) need to be further investigated using intraoperative designs and histological studies.

### Active force measurements

The active force-ankle angle characteristics shown presently (Figure 6) are consistent with the findings of previous studies in animal and human muscles (Gordon et al., 1966; Dulhunty and Franzini-Armstrong, 1977; Rassier et al., 1999; Smeulders et al., 2004; Kreulen and Smeulders, 2008; Ateş et al., 2013). In contrast to the major difference in passive forces found between neurological and non-neurological patients, five patients with non-neurological conditions and CP showed a similar shape of the active force-angle curve, with maximal force production at their medium muscle lengths (15°–45° ankle angle) and spanning both ascending and descending limbs of the curve. The only distinct characteristic was observed for the force excursion curve of the HMSN-patient performing at the descending limb only (with a maximum force at 60° PF). No published data is showing active mechanical characteristics of human lower leg muscles in health however, previous studies reporting the force-knee angle characteristics of hamstring muscles of patients with CP indicated a qualitative similarity to the shape of the active force curve of healthy hamstrings (Ateş et al., 2013; Ates et al., 2016; Kaya et al., 2019). In agreement with these, our present results indicate a comparably wide range of active muscle force production for patients with CP pre-ATL. As maximal force production was achieved around the optimal muscle lengths where the medium passive force was generated, a good overlap between muscle filaments (Gordon et al., 1966) is anticipated pre-ATL. This might mean that the affected muscle *per se* does not necessarily need to be lengthened for a wide range of active force production. The key to improved joint movement would be a shift of optimal muscle length to DF positions as observed post-ATL but with a minimal loss in active force production (Delp and Zajac, 1992). Previous studies reported major active force reductions (Ates et al., 2013) due to aponeurotomy and muscle lengthening surgeries since these interventions directly interfere with the force-transmitting components of muscles by cutting the connection of muscle fibers to the tendon through neighboring muscle fibers and aponeurosis (Yucesoy et al., 2013). Therefore, the surgery targeting the tendon for lengthening rather than the muscle itself is supported to be a better solution for the targeted patient group.

The mechanical changes induced by the ATL have multiple effects on patients’ gait. First, increased maximal dorsiflexion in the stance and swing phases leads to improved ankle kinematics (Hemo et al., 2006; Ma et al., 2021). This effect can be attributed to the reduction in tendon tension and restored dorsiflexion. In addition to the changes in kinematics, there are evident improvements in kinetics (ankle moment and power) (McMulkin et al., 2016; Putz et al., 2018) which could be explained by the ability of related muscles to generate force over a greater ROM post-ATL due to increased tendon length. Hence, the increase in dorsiflexion and a wider range of force generation could lead to better walking ability reported after ATL procedures (Hemo et al., 2006; McMulkin et al., 2016; Putz et al., 2018; Ma et al., 2021).

### Feasibility and clinical relevance of intraoperative measurements

In the tendon lengthening surgery, as presented, objective force data can help to determine the best position for the refixation of the tendons with the right tension to minimize the loss in active force production (Lieber et al., 1996). The implementation of this new technique in surgery routines can initially be challenging. However, once employed, it is a straightforward method that provides surgeons with immediate objective information. Passive data can be collected within 5 minutes. Active force data collection, however, can take about 20 min as the muscle needs recovery periods between consecutive measurements. Though, the active force data is still relevant since with the present knowledge, the passive force cannot be used directly as a predictor of the characteristics and magnitude of active force production. For this to be feasible, there is a need for more data to be collected and reliable muscle models that are validated for specific conditions and diseases.

Both passive and active force production showed high inter-subject variability, even within the same group of patients. Until further research and results on the etiology and pathophysiology of muscle shortening are available to allow a more precise classification, force data should be collected for each patient individually. Particularly for patients with idiopathic foot deformities, this study provides important results on force production and tendon tension that can be further built upon.

More importantly, the presented method is not necessarily limited to the measurement of triceps surae forces. A similar approach can be used, for example, to measure tibialis anterior and tibialis posterior muscle forces during tendon transfers. Therefore, in tendon transfer and muscle lengthening surgery, the surgeon’s intuition and experience can be supplemented with objective force data. Consequently, our findings suggest that the method improved in this study 1) can be used to determine the new optimal muscle length and 2) would provide comparability and thus improve surgical outcomes.

## Data Availability

The raw data supporting the conclusion of this article will be made available by the authors, without undue reservation.
